# Hydrocephalus Complicating Intrathecal Antisense Oligonucleotide Therapy for Huntington's Disease

**DOI:** 10.1002/mds.28359

**Published:** 2020-10-30

**Authors:** Thomas B. Stoker, Katie E.R. Andresen, Roger A. Barker

**Affiliations:** ^1^ John van Geest Centre for Brain Repair, Department of Clinical Neurosciences University of Cambridge Cambridge United Kingdom; ^2^ Wellcome Trust–Medical Research Council Stem Cell Institute University of Cambridge Cambridge United Kingdom

Huntington's disease is a genetic disorder caused by an expanded CAG repeat in the *huntingtin* gene, and although there are currently no disease‐modifying treatments, there is much excitement about the prospect of treatments targeting huntingtin expression. In a phase I/2A trial of an antisense oligonucleotide (ASO) treatment (tominersen), no serious adverse events were recorded, and there was a dose‐dependent reduction in cerebrospinal fluid (CSF) huntingtin levels.^1^ In an open‐label extension (OLE) study, patients received monthly or bimonthly tominersen, with preliminary data confirming the reduction in mutant huntingtin levels.^2^ Here we report on a unique major adverse effect occurring during this OLE.

A 54‐year‐old man with a pathogenic *huntingtin* CAG repeat of 42 received monthly intrathecal tominersen during the OLE, having received four doses of intrathecal placebo during the prior phase I/2A trial. At the start of the OLE he had modest chorea and broken ocular pursuit, with a total Unified Huntington's Disease Rating Scale motor score of 12. His mobility was normal, he performed tandem gait without support, and he was still working.

Following his fifth monthly dose of tominersen, he experienced gait difficulties, with an unsteady broad‐based gait along with mild finger‐nose and heel‐shin ataxia and an inability to perform a tandem gait. He initially continued to receive monthly tominersen, but his gait deteriorated over the following 3 months; he started to fall and could no longer work. His Unified Huntington's Disease Rating Scale motor score increased to 38.

His clinical deterioration was accompanied by a progressive increase in CSF protein, peaking at 2.64 g/L, and a CSF lymphocytosis, peaking at 46 cells/mm^3^ (Fig. 1A,B). Serial brain magnetic resonance imaging revealed increasing ventricular dilation, with periventricular edema, consistent with hydrocephalus (Fig. 1C,D). His gait improved dramatically after a high‐volume CSF tap, and lumbar infusion studies confirmed increased resistance to CSF outflow (Fig. 1E). A ventriculoperitoneal shunt was therefore inserted, and his mobility improved to his baseline state. He did not receive any further doses of tominersen.

**FIG. 1.**
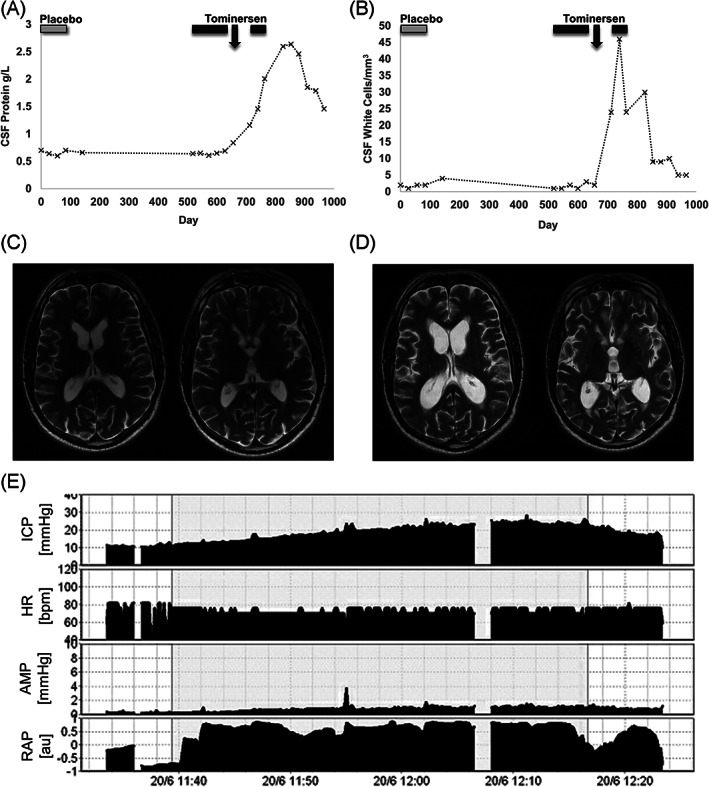
Investigation results. Cerebrospinal fluid (CSF) protein (**A**) and leukocyte count (**B**). Light and dark gray bars represent treatment with placebo and tominersen, respectively. Arrow indicates clinical deterioration. Axial T2‐weighted magnetic resonance imaging at baseline (**C**) and after deterioration (**D**) Lumbar infusion study (**E**) AMP, pulse amplitude; HR, heart rate; ICP, intracranial pressure; RAP, compensatory reserve index.

This ASO therapy has led to much excitement within the Huntington's disease community, but the published clinical data are limited, with no major serious adverse events reported. Here we report for the first time on a patient in receipt of this therapy who developed a communicating hydrocephalus that we diagnosed as being secondary to a sterile meningitis induced by tominersen. This resulted in significant disability, which benefited from shunting.

Clinical experience with intrathecal ASO therapy is limited but does include the use of the intrathecal ASO treatment (nusinersen) for spinal muscular atrophy.^3^, ^4^ Although no cases of hydrocephalus were seen in trials of this drug, during postmarketing surveillance a number of cases of aseptic meningitis and communicating hydrocephalus were observed.^5^ However, this has not been reported with tominersen to date and given that a large phase 3 study is underway (NCT03761849), along with other similar studies,^6^ it is important that this potential complication is recognized.

## Author Roles

T.B.S.: Preparation of manuscript.

K.E.R.A.: Review and critique of manuscript.

R.A.B.: Review and critique of manuscript.

## Financial disclosures

R.A.B. is supported by the National Institute for Health Research (NIHR) Cambridge Biomedical Research Centre (146,281; the views expressed are those of the author(s) and are not necessarily those of the NIHR or the Department of Health and Social Care) and MRC/WT Stem Cell Institute (203,151/Z/16/Z).

Ethical approval for the trial in which this patient participated was granted by London–West London & GTAC Research Ethics Committee, and the REC reference number was 17/LO/1502. The patient provided written consent to take part, and the patient provided signed written consent for publication of this report. We confirm that we have read the Journal's position on issues involved in ethical publication and affirm that this work is consistent with those guidelines.
